# Cooperative Search Method for Multiple UAVs Based on Deep Reinforcement Learning

**DOI:** 10.3390/s22186737

**Published:** 2022-09-06

**Authors:** Mingsheng Gao, Xiaoxuan Zhang

**Affiliations:** College of Internet of Things, Hohai University, Changzhou 213002, China

**Keywords:** task assignment, multi-UAV, deep reinforcement learning

## Abstract

In this paper, a cooperative search method for multiple UAVs is proposed to solve the problem of low efficiency of multi-UAV task execution by using a cooperative game with incomplete information. To improve search efficiency, CBBA (Consensus-Based Bundle Algorithm) is applied to designate the tasks area for each UAV. Then, Independent Deep Reinforcement Learning (IDRL) is used to solve Nash equilibrium to improve UAVs’ collaborations. The proposed reward function is smartly developed to guide UAVs to fly along the path with higher reward value while avoiding the collisions between UAVs during flights. Finally, extensive experiments are carried out to compare our proposed method with other algorithms. Simulation results show that the proposed method can obtain more rewards in the same period of time as other algorithms.

## 1. Introduction

Nowadays, unmanned aerial vehicles (UAVs), or drones, are widely used in the witness of a fast-paced development [1]. Equipped with radar, cameras and other equipment, UAVs can be used in military areas [2] such as for tracking, positioning and battlefield detection. However, due to the limitation of fuel load, it is difficult for a single UAV to search a large area. Compared with a single UAV, multiple UAVs can perform more complex tasks. Multiple UAVs sharing information and searching cooperatively can improve the efficiency of task execution. In the process of the search task, the path planning of the multi-UAV is a crucial problem [3].

For the above problem, many scholars have proposed some multi-UAV path planning algorithms. For instance, hierarchical decomposition is one of the effective way to solve the problem. The clustering algorithm is first used for the multi-UAV task assignment. Then the path planning is based on the Voronoi diagram [4] or genetic algorithm [5]. However, these path planning algorithms require a prior knowledge about the environment and centralized task assignment algorithms require a control center to communicate among UAVs, which is not suitable in dynamic scenarios. On the other hand, multi-agent reinforcement learning (MARL) is effective to solve the above problem. The essence of MARL is a stochastic game. MARL combines the Nash strategies of each state into a strategy for an agent while constantly interacting with the environment to update the Q value function in each state of the game. Nash equilibrium solution in MARL can replace the optimal solution to obtain an effective strategy [6].

In this paper, we explicit Independent Deep Reinforcement Learning (IDRL) to solve the problem of low efficiency when multiple UAVs perform tasks simultaneously. CBBA [7] (Consensus-Based Bundle Algorithm) is first used for task assignment for multiple UAVs under constraints of time and fuel consumption. Then the UAV chooses the best strategy to complete the task based on the states and actions of other UAVs. A new reward function is developed to guide the UAV to choose the path with high value and punish collisions between UAVs.

The main contributions of this paper are summarized as follows:

(1) Different from the centralized path planning algorithm that a central controller is required for task allocation, a distributed path planning algorithm is designed in this paper. UAVs can communicate with each other for task allocation and path planning in a more flexible way.
(2) A cooperative search method is proposed. Before selecting the next action, the UAV needs to adopt the corresponding cooperative method according to the incomplete information obtained to improve the efficiency of task execution.
(3) A new reward function is proposed to avoid collisions between UAVs while guiding UAVs to target points.

## 2. Related Work

In this section, we review the literature that settles multi-UAV target assignment and path planning (MUTAPP), figure out their pros and cons and clarify the remaining gaps and challenges for further investigations.

MUTAPP problem is an NP-hard problem in essence, which implies there is no perfect solution to an NP-hard problem. However, for small- and/or medium-sized problems, it is possible to be solved. Hierarchical decomposition is one of the effective methods to solve MUTAPP [8], which decomposes the MUTAPP problem into task assignment and path planning.

At present, MUTAPP is mainly divided into traditional MTSP and objective function optimization problems. For traditional MTSP, Wang et al. [9] try to use genetic algorithms for task assignment and cubic spline interpolation for path planning. In [10], Liu et al. use Overall Partition Algorithm (OPA) for task assignment and use cycle transitions to generate shortest paths. Simulation results show that the proposed algorithm achieves better performance than traditional algorithms based on GA to solve MTSP problems. In [11], Dubins curves are used to model the UAV kinematics model to make the generated path more realistic. The improved particle swarm optimization algorithm based on heuristic information is proposed to solve MTSP. The results show that the proposed algorithm can generate paths in a small number of iterations. However, in practical applications, the multi-UAV system not only needs to consider the total flight distance, but also the efficiency of the task which is usually evaluated by the objective function.

With a single UAV and no altitude effects, the standard coverage path planning (CPP) problem has been studied extensively in the literature [12,13]. The objective function of CPP is defined as the area of the covered region. Miles et al. [14] proposes rectangle partition relaxation (RPR) algorithm to divide the UAV flight area. In [15], based on the single UAV algorithm, a density-based sub-region of UAV coverage with a unique role is proposed to optimize the coverage area. Xie et al. [16] provides a mixed-integer programming formula for CPP and develops two algorithms based on this method to solve the TSP-CPP problem. Based on this research, Xie extends the proposed algorithm in [17] and proposes a branch-and-bound-based algorithm to find the optimal route. Although these algorithms continuously optimize the UAV coverage area, it is difficult to evaluate the efficiency of UAV execution with a single constraint.

In [18], the objective function of the multi-UAV system is to minimize energy loss. K-means is used to assign tasks to multiple UAVs, and then genetic algorithm is used to generate specific paths. In [19], simulated annealing algorithm is used to increase the coverage area of the UAV. Reference [18] uses the more advanced k-means++; the experimental results show that the generated path is shorter than k-means. In [20], the Minimum Spanning Tree (MST) is used to generate trajectories and simulation results show that compared with other algorithms, the generated trajectories can obtain more rewards during task execution. Also, there are some algorithms [21,22,23,24,25] that use clustering algorithm to solve MUTAPP related problems. However, clustering algorithm is sensitive to noise points. If the task point is far from the central point, it will be assigned to the UAV separately, which is unrealistic.

Wang et al. [26] use MST to decompose MTSP into multiple TSPs, and then Ant colony algorithm is used to solve TSP. In [27], a fuzzy approach with a linear complexity level is used to convert the MTSP to several TSPs, then Simulated Annealing (SA) is used to solve each problem. Similarly, Cheng et al. [28] decouples the MTSP problem into TSP and solves the subproblems through sequential convex programming. Reference [29] propose a task allocation algorithm based on maximum entropy principle (MEP). Simulation results show that the proposed MEP algorithm achieves better performance than SA algorithm. Cao et al. [30] introduces Voronoi diagram method into Ant colony algorithm and the unmanned aerial vehicle cooperative task scheduling strategy which conclude task allocation and path planning is gained. Compared with clustering algorithm, these algorithms are more flexible in task allocation and the number of tasks performed by each agent is reduced through reasonable task allocation, which increases the execution efficiency of the algorithm. However, since the cooperation among agents is not considered, which would affect the efficacy of these algorithms.

MARL provides a new solution for MUTAPP problems; it models the decision-making process in the multi-agent environment as a random game where each agent needs to make decisions according to the strategies of other agents. MARL has become a prevalent method to solve the problem of multi-agent cooperation.

In [31], DRL is used to generate paths for data collected by multiple UAVs without prior knowledge. Reference [32] uses MADDPG for the cooperative control of four agents; the experimental results show that MADDPG has good performance in complex environments and successfully learns the strategy of multi-agent collaboration. However, with the instability of the environment caused by the increase in the number of agents, the proposed algorithm has certain difficulties in the joint action space. In [33], MADDPG is used to control the formation of multiple agents during transportation in order to prevent the agent from colliding with other agents on the way to the target point. Chen et al. [34] use MARL for the collaborative welding of multiple robots. The way of cooperation between robots is also to prevent collisions between agents.

Han et al. [35] use MADDPG for both task assignment and path planning, and a reward value function is designed to guide the UAV to the target point and avoid collisions between UAVs. In fact, the cooperative approach of avoiding conflict can improve the success rate of task execution but does not directly affect the efficiency of task execution. Also, the proposed algorithm only works in environments where each agent performs one task and cannot be used to solve the multiple traveling salesman problem. Moreover, the performance of value-based reinforcement learning is better than that of policy-based reinforcement learning in the task environment with few actions.

## 3. Overview of CBBA

In this section, we will review the CBBA algorithm, which is generally divided into two parts: bundle construction and conflict resolution.

### 3.1. Bundle Construction

In the process, each CBBA agent creates only one bundle and updates it during the allocation process. In the first phase of the algorithm, each agent keeps adding tasks to its bundle set until no other tasks can be added.

During the task assignment process, each agent needs to store and update the following four necessary information vectors: a bundle $b_{i}\in {\left(J\cup^{\;}\left\{\emptyset \right\}\right)}^{L_{t}}$, the corresponding path $p_{i}\in {\left(J\cup^{\;}\left\{\emptyset \right\}\right)}^{L_{t}}$, the winning agent list $z_{i}\in J^{N_{t}}$ and the winning score list $y_{i}\in R_{+}^{N_{t}}$.

The sequence of tasks in the bundle is arranged according to the order in which the tasks are added to the collection, and the tasks in the path are arranged according to the order in which the tasks are best executed. Note that the vector size of $b_{i}$ and $p_{i}$ cannot be greater than the maximum assigned task number $L_{t}$. $S_{i}^{p_{i}}$ is defined as the total reward score value of the task i performing the task along the path $p_{i}$. In CBBA, adding task *j* to bundle $b_{i}$ will result in an increase in marginal scores:

(1) $$c_{ij}\left[b_{i}\right]=\{\begin{array}{l} 0,\;\;\;\;\;\;\;\;\;\;\;\;\;\;\;\;\;\;\;\;\;\;\;\;\;\;\;\;\;\;\;\;\;\;\;\;\;\;\;\;\;\;\;\;\;\;\;\;\;\;\;\;\;if\;j\in b_{i}\\ \underset{n\leq \left|p_{i}\right|+1}{\max}S_{i}^{p_{i}{⊕}_{n}\left\{j\right\}}-S_{i}^{p_{i}},\;\;\;\;\;\;\;\;\;\;\;\;\;\;\;otherwise \end{array}$$

where |·| represents the vector size of the list, ${⊕}_{n}$ represents the insertion of a new element after the n-th element of the vector (in the later part of this article, ${⊕}_{end}$ will also be used to indicate the addition of a new element at the end of the vector). CBBA’s bundle scoring scheme inserts a new task into the position where the highest score increases, which will be the marginal score associated with the task in a given path. Therefore, if the task is already included in the path, there will be no extra scores.

The score function is initialized as $S_{i}^{\left\{\emptyset \right\}}=0$, and the path and bundle are recursively updated to

(2) $$\;\;\;b_{i}=b_{i}{⊕}_{end}\left\{J_{i}\right\},p_{i}=p_{i}{⊕}_{n_{i},J_{i}}\left\{J_{i}\right\}\;\;\;$$

where $J_{i}=argmax_{j}(c_{ij}\left[b_{i}\times h_{ij}\right)$, $n_{i,J_{i}}=argmax_{n}S_{i}^{p_{i}{⊕}_{n}\left\{J_{i}\right\}}$, $h_{ij}=II\left(c_{ij}>y_{ij}\right)\;and\;II\left(\cdot \right)$ indicates an index function that having a value of 1 when the judgment result is true and a value of 0 when the judgment result is false. The bundle algorithm is continuously looped until $\left|b_{i}\right|=L_{t}$ or $h_{i}=0$.

### 3.2. Conflict Resolution

In the conflict resolution phase, there are three aspects that need to be communicated to reach a consensus. The two vectors that have been introduced are the winning score list $y_{i}\in R_{+}^{N_{t}}$ and the winning agent list $z_{i}\in J^{N_{t}}$. The third vector $s_{i}\in R^{N_{u}}$ represents the timestamp of the last information update from each other agent. The time vector is updated by:

(3) $$s_{ik}=\{\begin{array}{c} \tau_{r},\;\;\;\;\;\;\;\;\;\;\;\;\;\;\;\;\;\;\;\;\;\;\;\;\;\;\;\;\;\;\;\;\;\;\;\;\;\;\;if\;g_{ij}=1\\ \underset{m:g_{im}=1}{\max}s_{mk},\;\;\;\;\;\;\;\;\;\;\;\;\;\;\;\;\;\;\;\;\;\;otherwise \end{array}$$

where $\tau_{r}$ is the message reception time.

When agent *i* receives a message from agent *k*, $z_{i}$ and $s_{i}$ are used to determine the information of which agent in each task is up to date. For task *j*, agent *i* has three possible actions:

1. Update: $y_{ij}=y_{kj},z_{ij}=z_{kj}$
2. Reset: $y_{ij}=0,z_{ij}=\emptyset$
3. Leave: $y_{ij}=y_{ij},z_{ij}=z_{ij}$

Table 1 in [7] outlines the decision rules for information interaction between agents.

If the elements in the winning score list change due to communication, each agent will check whether the updated or reset tasks were in their bundle. If the task is actually in the bundle, then this task and all other tasks added to the bundle later will be released:

(4) $$\;\;\;y_{i,b_{in}}=0,z_{i,b_{in}}=\emptyset ,\;\forall n>\bar{n_{l}}$$

(5) $$b_{in}=\emptyset ,\;n\geq \bar{n_{l}}$$

where $b_{in}$ represents the ***n***-th element of the bundle, and $\bar{n_{l}}=\min\left\{n:z_{i,b_{in}}\neq i\right\}$. It should be noted that the task that adding to the winning agent and the winning list after $b_{i,\bar{n_{l}}}$ will be reset because the deletion can change all the task scores after $b_{in}$. After completing the second phase of conflict resolution, the algorithm will return to the first phase and add a new task.

## 4. IDRL Based Path Planning Algorithm

Independent Reinforcement Learning (IRL) is widely and successfully applied in the field of multi-agent autonomous decision-making. This paper uses IDRL to solve Nash equilibrium in a cooperative game with incomplete information, and each UAV chooses the optimal strategy according to the states and actions of other UAVs to maximize the total rewards.

### 4.1. System Model

In this paper, we establish a model based on IDRL to enhance the efficiency of task execution through multi-UAV cooperation. We make the following assumptions:

(1) Any two UAVs with intersected flight paths can communicate with each other to know the states and actions when the distance between them is less than a threshold. The game between UAVs belongs to incomplete information games.
(2) Each UAV can choose the optimal strategy according to the state and action of other UAVs, so the game between UAVs belongs to cooperative games.
(3) The UAVs do not choose actions at the same time, so the game between UAVs belongs to dynamic games.

**Figure 1:**
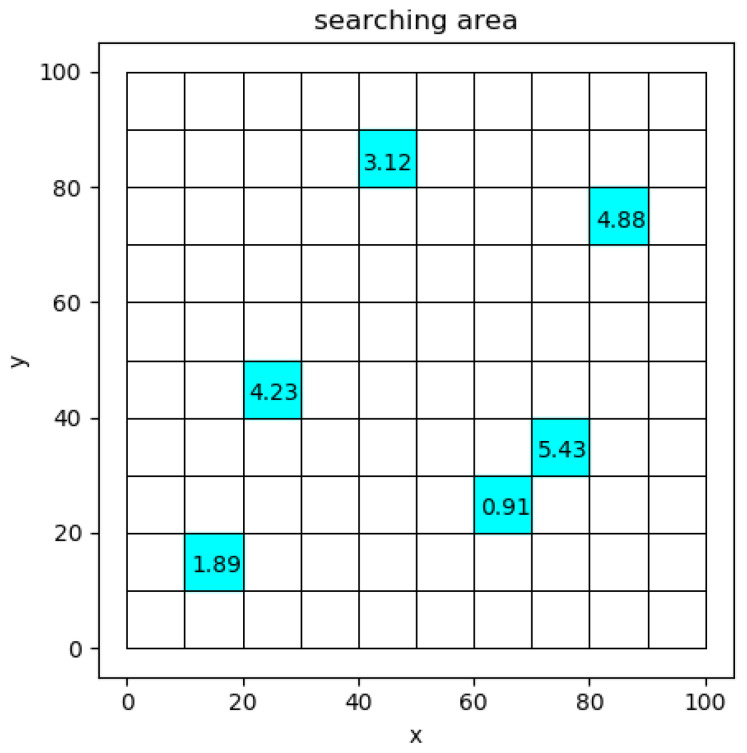
Grid partition of a searching area.

The task environment of multiple UAVs is briefly divided into two-dimensional grids, as shown in Figure 1. The blue part represents the task area to be executed.

In the cooperative game with incomplete information, the objective function of UAVs is to maximize the search efficiency. ROR and revenue defined in [36] are used to evaluate the search efficiency of UAVs.

The detection function is used to estimate the detection ability in a probable target grid j with time consumption z. A common exponential form of regular detection function is given as:

(6) $$\;b\left(j,z\right)=1-e^{-\varepsilon z}$$

where ε is a parameter related to the UAV equipment, z represents time consumption.

When an UAV is searching in grid *j*, the revenue function defined as

(7)   e(j,z)=p(j)b(j,z)

where *p*(*j*) represents the target probability in grid  j.

The efficiency of a multi-UAV system to perform a search task is assessed by the amount of reward per unit of time earned by multiple UAVs. Therefore, the ROR of grid j is introduced with a definition as:

(8) $$\;\;ROR\left(j,z\right)=\frac{d\left(e\right)}{d\left(z\right)}=\varepsilon \cdot e^{-\varepsilon z}\cdot p\left(j\right)b\left(j,z\right)$$

where indicates that the ROR value decreases as the search time *z* increases. In other words, a lower ROR indicates that the area is searched more thoroughly.

We assume that each UAV knows the ROR value of all grids, as shown in Figure 1. The problem can be solved into strategies on CBBA and DRL.

### 4.2. Nash Equilibrium in MARL

In MARL, $V_{i}\left(\pi_{1},\cdot \cdot \cdot ,\pi_{i},\cdot \cdot \cdot ,\pi_{n}\right)$ represents the expected reward of i-th agent under the joint strategy $\left(\pi_{1},\cdot \cdot \cdot ,\pi_{i},\cdot \cdot \cdot ,\pi_{n}\right)$. In a matrix game, if the joint strategy satisfies Equation (9), then the strategy is a Nash equilibrium.

(9) $$\;\;V_{i}\left(\pi_{1}^{*},\cdot \cdot \cdot ,\pi_{i}^{*},\cdot \cdot \cdot ,\pi_{n}^{*}\right)\geq V_{i}\left(\pi_{1}^{*},\cdot \cdot \cdot ,\pi_{i},\cdot \cdot \cdot ,\pi_{n}^{*}\right)$$

The essence of MARL is a stochastic game. MARL combines the Nash strategies of each state into a strategy for an agent and constantly interacts with the environment to update the Q value function in each state of the game.

The random game consists of a tuple $\;\langle N,S,{\left\{A^{i}\right\}}_{i\in \left\{1,\ldots ,N\right\}},P,\gamma ,{\left\{R^{i}\right\}}_{\left\{1,\ldots ,N\right\}}\rangle$, N represents the number of agents, S is the state space of the environment, and $A^{i}$ is the action space of agent i, P is the probability matrix of state transition, $R^{i}$ is the reward function of agent *i*, and *𝛾* is the discount factor. For multi-agent reinforcement learning, the goal is to solve the Nash equilibrium strategy in each stage game and combine these strategies.

The optimal strategy of multi-agent reinforcement learning can be written as $\left(\pi_{1}^{*},\cdot \cdot \cdot ,\pi_{n}^{*}\right)$ and for ∀s∈S, i=1,···,n, it have to satisfy Equation (10).

(10) $$\;\;V_{i}\left(s,\pi_{1}^{*},\cdot \cdot \cdot ,\pi_{i-1}^{*},\pi_{i}^{*},\pi_{i+1}^{*},\cdot \cdot \cdot ,\pi_{n}^{*}\right)\geq V_{i}\left(s,\pi_{1}^{*},\cdot \cdot \cdot ,\pi_{i-1}^{*},\pi_{i},\pi_{i+1}^{*},\cdot \cdot \cdot ,\pi_{n}^{*}\right)$$

$Q_{i}^{*}\left(s,a_{1},\cdot \cdot \cdot ,a_{n}\right)$ represents the action value function. In each phase game of state s, the Nash equilibrium strategy is solved by using $Q_{i}^{*}$ as the reward of the game. According to Bellman’s formula in reinforcement learning, MARL’s Nash strategy can be rewritten as Equation (11).

(11) $$\underset{a_{1},\cdot \cdot \cdot ,a_{n}}{\sum }Q_{i}^{*}\pi_{1}^{*}\cdot \cdot \cdot ,\pi_{i-1}^{*},\pi_{i}^{*},\pi_{i+1}^{*},\cdot \cdot \cdot \pi_{n}^{*}\geq \underset{a_{1},\cdot \cdot \cdot ,a_{n}}{\sum }Q_{i}^{*}\pi_{1}^{*}\cdot \cdot \cdot ,\pi_{i-1}^{*},\pi_{i},\pi_{i+1}^{*},\cdot \cdot \cdot \pi_{n}^{*}$$

In a random game, if the reward function of each agent is the same, the game is called complete cooperative game or team game. In order to solve the random game, stage game at each state s needs to be solved, and the reward obtained by taking an action is $Q_{i}\left(s\right)$.

### 4.3. Path Planning Algorithm Based on IDRL

#### 4.3.1. Environment States

In the cooperative game, UAVs need to choose the optimal strategy according to the state and action of other UAVs. Thus, at timestep *k*, the state vector of the *j*-UAV is represented by:

(12) $$\;s_{k}^{j}={\left[x_{j},y_{j},x_{1},y_{1},x_{t1},y_{t1},a_{1},\rho \right]}^{T}$$

where $x_{j}$ and $y_{j}$ represent the abscissa and ordinate of the ***j***-UAV, respectively. $x_{1},y_{1}$ represent the coordinates of the nearest UAV, $a_{1}$ represents the action of the nearest UAV at timestep ***k***. $x_{t1},y_{t1}$ represent the current task coordinates of ***j***-UAV. The value of ρ is 0 or 1, indicating whether the area surrounding the UAV has been searched by other UAVs.

#### 4.3.2. Discrete Action Set

**Figure 2:**
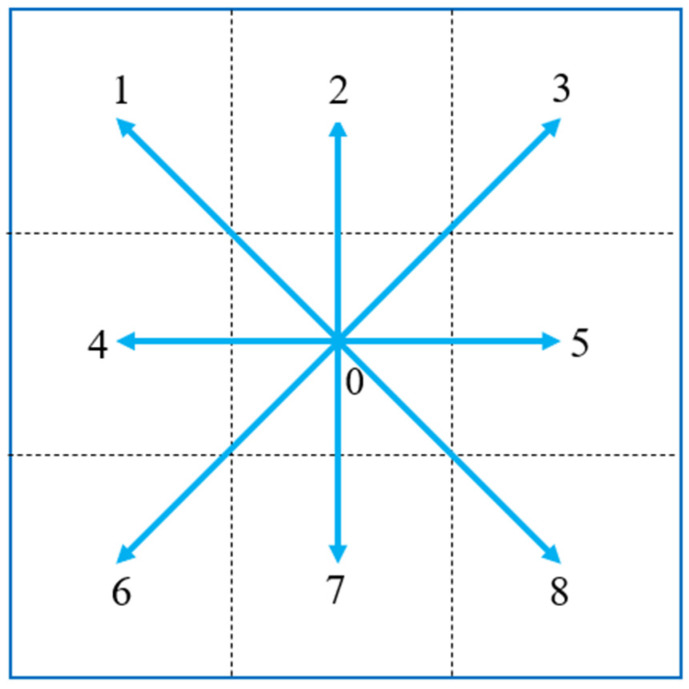
The action set of the UAV. The numbers represent the numbers of the 9 grids respectively.

Since the length of the grid in the task environment is much larger than the turning radius of the UAV, it can be assumed that the UAV moves in a straight line in the grid. As shown in Figure 2, when the UAV is in the grid 0, it can perform eight actions to go to the corresponding grid. The numbers in the grids represent eight actions, including: left up, up, right up, left, right, left down, down, and right down.

#### 4.3.3. Reward Function

The reward function is used to evaluate the quality of the action. In fact, there are many factors that could affect the action selection of UAV, but within the scope of research, the following three factors are mainly considered:

- Choosing the shortest path to the destination.
- Encouraging actions passing high ROR areas.
- Preventing collisions between UAVs.

Choosing the shortest path to the target area is not always optimized for path planning, but still has a very high priority in the process. In order to prevent the reward value from being too sparse and speed up the convergence of the IDRL algorithm, a continuous reward function is proposed for the discrete environment. The reward for taking the shortest path is formulated as follows:

(13) $$\;\;R_{1}=\{\begin{array}{c} \;\;\;\;\;\;\;\;\;\;\;\;40\;\;\;\;\;\;\;\;\;\;\;\;\;\;\;\;\;\;\;\;\;\;\;\;\;\;\;\;\;\;\;\;\;\;\;\;\;\;\;\;\;\;\;\;\;\;\;if\;end\;\;\;\;\;\;\\ \frac{100}{d_{t}}*10^{\left(-y\right)}\;\;\;\;\;\;\;\;\;\;\;\;\;\;\;\;\;\;\;\;\;\;\;\;\;\;\;\;\;\;else \end{array}$$

In which, y is the integer that increases with the distance of UAV from the target point, $d_{t}$ is the current Euclidean distance from the UAV to the target point. We set the coordinates of j-UAV as ($x_{j},y_{j}$), and the coordinates of the target point as ($x_{2},y_{2}$), then

(14) $$d_{t}=\sqrt{{\left(x_{j}-x_{2}\right)}^{2}+{\left(y_{j}-y_{2}\right)}^{2}}$$

In the process of reward value learning, if the reward values of adjacent states are too close, the algorithm may fall into the trap of local optimization due to insufficient training samples. Therefore, for the discovery rate ε, the discovery rate is set to 0.4 to encourage exploration at the beginning of searching for the optimal path. When the algorithm tends to converge, the discovery rate should be reduced to make it approach 0.

**Figure 3:**
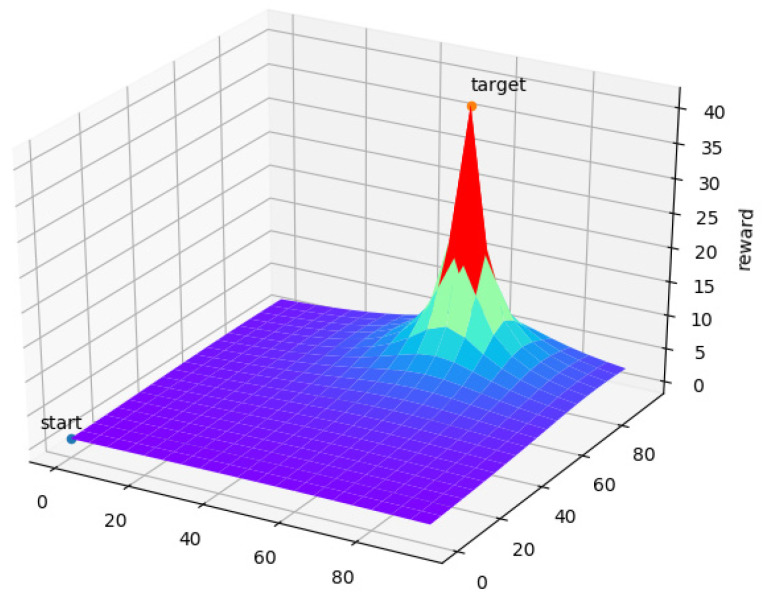
Image of reward function $R_{1}$.

The reward function of $R_{1}$ is shown in Figure 3. By using the reward function $R_{1}$, the UAV can choose the shortest path to the target point according to the reward value obtained.

When UAVs perform search tasks in the same area without a preset mode of cooperation, different UAVs may detect repeated messages, causing meaningless time loss. In addition, performing search tasks in the same area can easily lead to UAV collisions.

To prevent collisions between UAVs, we add a small penalty when the distance between two UAVs is less than ($2*\sqrt{2}*d_{g}$) in length.

(15) $$R_{2}=\{\begin{array}{l} -e^{-100d_{u}},\;\;\;\;\;\;d_{u}\leq 2*\sqrt{2}*d_{g}\\ 0,\;\;\;\;\;\;\;\;\;\;\;\;\;\;\;\;\;\;\;\;\;d_{u}>2*\sqrt{2}*d_{g}\\ -1,\;\;\;\;\;\;\;\;\;\;\;\;\;\;\;\;\;\;\;\;\;\;\;\;\;\;\;\;\;\;\;\;\;\;\;d_{u}=0 \end{array}\;\;\;\;\;\;\;\;\;\;\;\;\;\;\;\;\;\;\;\;\;\;\;\;\;\;\;\;\;\;\;\;\;\;\;\;\;\;\;\;\;\;\;$$

where $d_{u}$ is the minimum distance between the i-th UAV and the nearest UAV. $d_{g}$ is the length of the grid in the task model.

**Figure 4:**
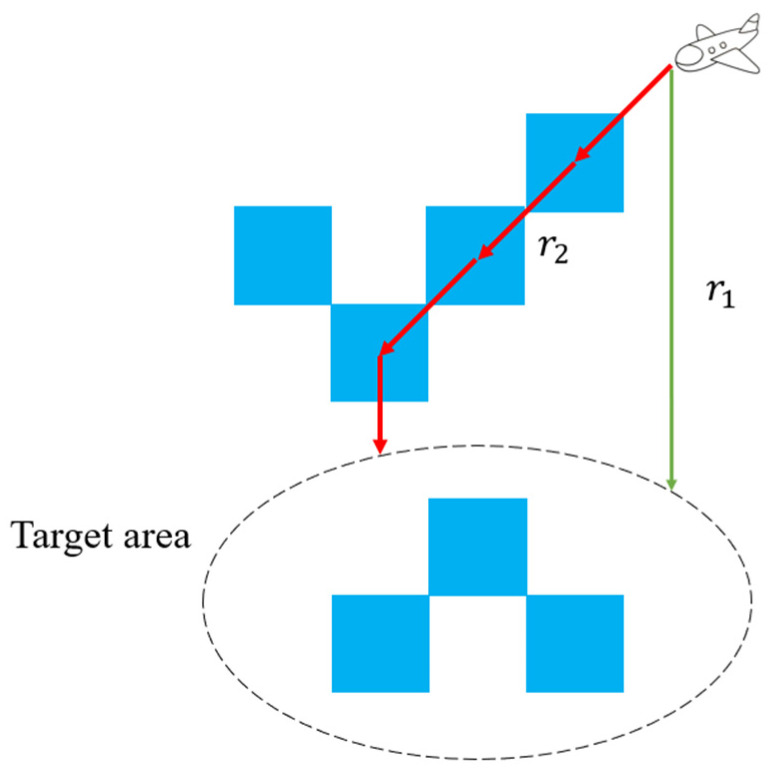
Path planning.

**Figure 5:**
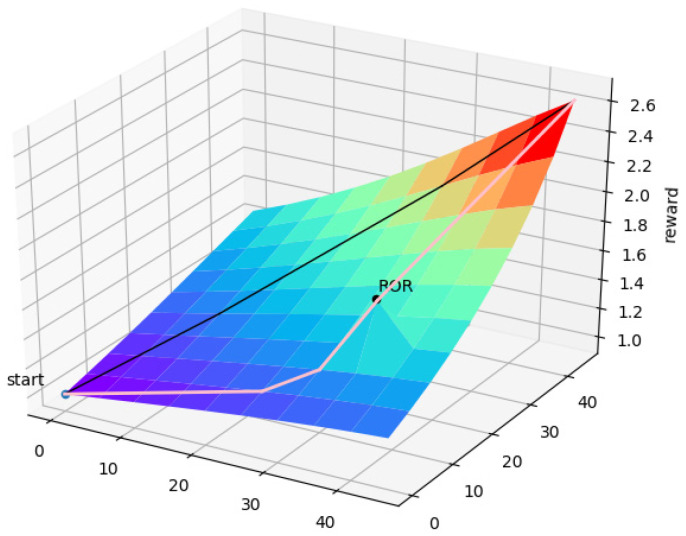
The combination of reward functions $R_{1}$ and $R_{3}$.

When an UAV flies to the assigned target area, the UAV needs to choose a reasonable path. Specifically, UAVs need to make decisions before moving to target areas. As shown in Figure 4, $r_{1}$ is the shortest path for the UAV to the target area. If the path is always the shortest route, the UAV will sometimes miss the target grids with high ROR values. Compared with the path $r_{1}$, the path $r_{2}$ is a more reasonable path. In order to improve the efficiency of UAVs to perform search tasks, the reward function needs to guide the UAV to the target point while passing through the high ROR area on the way. Thus, the reward function $R_{3}$ is related to the ROR value of each grid. The combination of reward functions $R_{1}$ and $R_{3}$ is shown in Figure 5.

When the reward function $R_{1}$ is used to train the UAV, the UAV will choose the straight path. When $R_{1}$ is combined with $R_{3}$, the UAV will choose the detour path and will not fall into the local optimum.

However, when a task area has been searched by UAV, it will waste time for other UAVs to search this area again, so it is more reasonable to choose path $r_{1}$. Therefore, UAV needs to decide which path to choose according to the following formula:

(16) $$\;\;R_{3}=\{\begin{array}{c} 0,\;\;\;\;\;\;\;\;\;\;\;\;\;\;\;\;\;\;\;\;\;\;if\;\rho =1\;\;\;\;\;\;\;\;\;\;\;\;\;\\ ROR,\;\;\;\;\;\;\;\;\;\;\;\;\;\;\;\;if\;\rho =0\;\;\;\;\;\;\;\;\;\;\;\;\; \end{array}$$

Therefore, the final reward value function is the sum of the reward values of all parts, each part of the reward value multiplied by an appropriate coefficient.

(17) $$\;\;\;\;\;R_{total}=\overset{3}{\underset{i=1}{\sum }}R_{i}*k_{i}\;\;$$

where $k_{i}$ is the coefficient for rewarding of each reward.

These coefficients represent the proportion of importance of each reward, which can be different between UAVs. Variation of these coefficients could alternate the output results. For example, getting more rewards can use a high value for the coefficient of $R_{3}$, while avoiding collisions that can use a low value for the coefficient of $R_{2}$.

## 5. Experiments and Discussions

In this section, we build two simulated search task environments, with 16 points and 29 points, respectively. Experiments are carried out using the above method as well as other algorithms.

### 5.1. Simulation Environment

**Figure 6:**
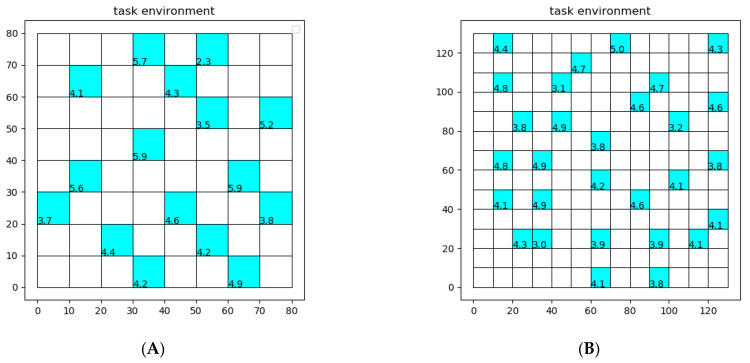
Task environment of two maps. (**A**) task environment of Map (a). (**B**) task environment of Map (b).

Map (a) in Figure 6A is 80 × 80 ($m^{2}$) while Map (b) in Figure 6B is 130 × 130 ($m^{2}$). The coordinate and ROR of each task area in two maps are shown in Table 1 and Table 2.

### 5.2. Parameters Setting

The parameter Settings of the experiment are shown in Table 3. In the experiment, parameters of CBBA and IDRL need to be set, respectively. For CBBA, the maximum number of tasks each UAV can carry out is 9. There is no termination time for each task, and the end condition of the UAV search task in each area is that the ROR of the current task area is less than 0.15 times the initial ROR of the task area.

For IDRL, the number of iterations of UAV is 20,000 times. When each UAV completes its task, it stops moving and communicating.

### 5.3. Results and Discussions

We first compare our proposed algorithm with *k*-means algorithm and minimum spanning tree algorithm in the same simulation environment.

**Figure 7:**
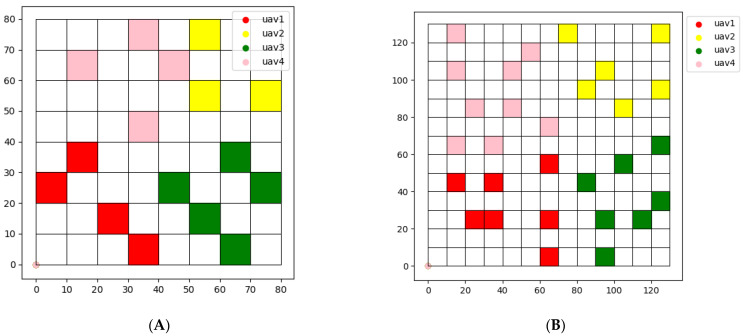
Task assignment using clustering algorithm on two maps. (**A**) Map (a). (**B**) Map (b).

In terms of task allocation, the results of using the clustering algorithm on two maps are shown in Figure 7.

Compared with CBBA, the advantage of using clustering algorithm for task allocation is that the task areas of each UAV are concentrated, and the UAV will not collide with other UAVs during flight. However, the dispersed task area makes it difficult to cooperate among multiple UAVs.

**Figure 8:**
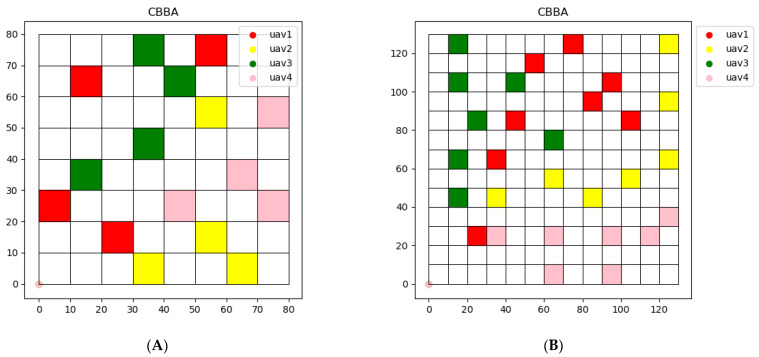
Task assignment using CBBA on two maps. (**A**) Map (a). (**B**) Map (b).

The result of using CBBA for task allocation is shown in Figure 8. For CBBA, since CBBA is essentially an auction algorithm, each UAV chooses tasks with the goal of maximizing rewards. Compared with clustering algorithm, the task area assigned by CBBA is more dispersed. At the same time, due to the consideration of time constraints, multiple UAVs can complete the tasks around the same time. The task completion time of each UAV using CBBA algorithm is shown in Table 4.

**Figure 9:**
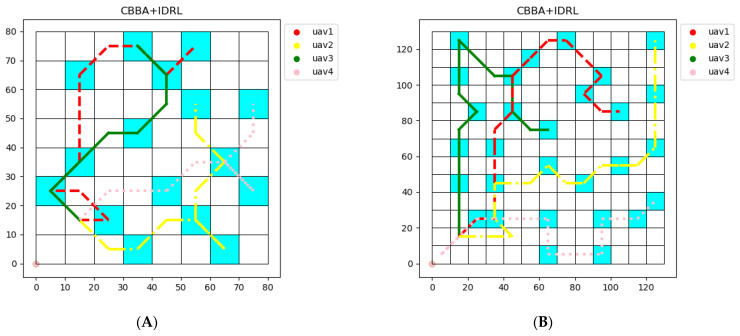
Paths generated using CBBA and IDRL. (**A**) Map (a). (**B**) Map (b).

However, the disadvantage of using CBBA for path planning is that UAVs are prone to collision and crash, and the cooperation between UAVs is not considered in CBBA. IDRL overcomes the above shortcomings. As shown in Figure 9, collisions between UAVs can be avoided by using IDRL and UAVs can choose the path with higher rewards.

**Figure 10:**
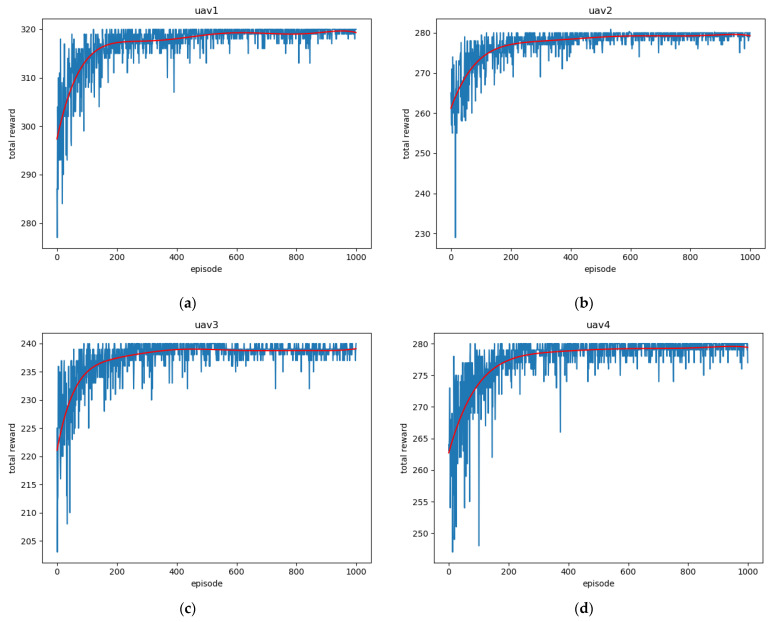
Reward value curves of four UAVs during training: (**a**) UAV1; (**b**) UAV2; (**c**) UAV3 (**d**) UAV4.

The reward value curves of UAVs in the training process are shown in Figure 10. The reward curve of the UAV in the training process represents the convergence of the algorithm. For map (b), with a more complex task model, as shown in Figure 9, the four agents will undergo a lot of trial and error at the beginning of training. The proposed algorithm is applied to map (b), convergence can be achieved in 1000 iterations, and the collision-free path can be formed eventually.

**Figure 11:**
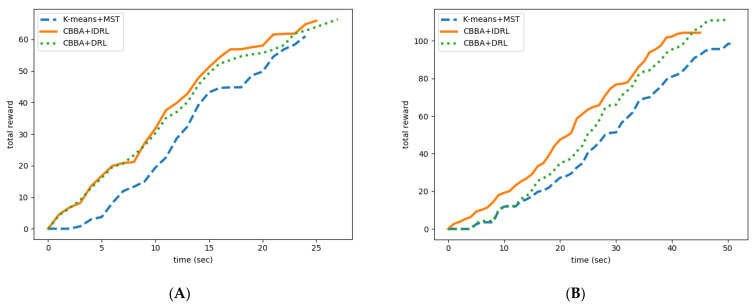
Total revenue variation in different algorithms. (**A**) Map (a). (**B**) Map (b).

The changing rules of total revenue of the different algorithms are shown in Figure 11. In two experimental environments, our proposed algorithm can obtain more rewards in the same time period than the K-means+MST algorithm. It is proved that our proposed algorithm has higher search efficiency. In addition, in order to prove that the proposed algorithm can improve the efficiency of search task execution through cooperation, we compared the proposed algorithm with the classical DRL algorithm under the premise that CBBA task assignment is also adopted. The results show that although DRL can get more reward value after completing the task, the time to complete the task is higher than IDRL and the reward value obtained by DRL is lower than IDRL in the same time. It is proved that IDRL can improve the efficiency of task execution through cooperation.

The residual ROR after simulations is shown in Table 5. For CBBA and IDRL, the ROR of most target grids can be reduced to a lower level due to the reasonable path optimization. Though some grids are also fully searched in another algorithm, there are more target grids with high ROR. The results show that our proposed algorithm can search the area more thoroughly.

**Figure 12:**
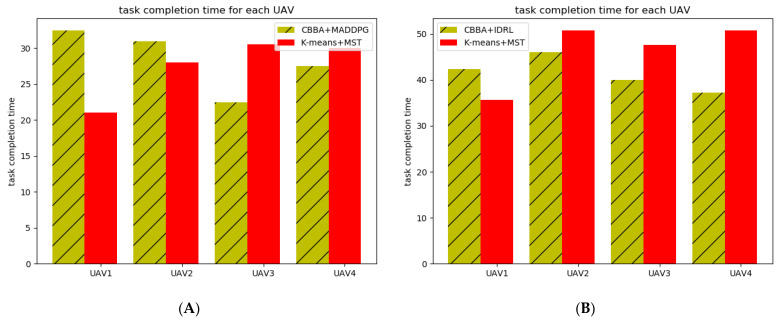
Task completion time for each UAV. (**A**) Map (a). (**B**) Map (b).

For a multi-UAV system, the time for each UAV to complete the task needs to be as short and close as possible. We compare the time for each UAV to complete the task between the two algorithms. As shown in Figure 12a, in small-scale scenarios, the proposed algorithm is close to the MST method in terms of time variance and mean value. However, in large-scale scenarios, our proposed algorithm shows obvious advantages, compared with k-means and MST algorithms, and the average time to complete the task with IDRL and the variance of the time to complete the task with four UAVs are smaller.

## 6. Conclusions

This paper first summarizes the existing path planning algorithms and points out their shortcomings. Then the search task model is introduced. On this basis, a cooperative search method of multiple UAVs is proposed. For task points with different reward values, CBBA is first used for task assignment. Then we use IDRL for UAV path planning and propose a new reward function. The proposed reward function consists of three parts, which are respectively used to guide UAV to the target point, avoid collision between UAVs and encourage UAV to choose the path with higher rewards. Experimental results show that compared with the other method, our proposed method can obtain more reward values in the same time and it is feasible and effective for multi-UAV path planning. In our future work, our focus will be on the constraints of the UAV kinematics by integrat-ing the Dubins curve model, which would make the proposed framework more practical.

## Figures and Tables

**Table 1:** Task area coordinates and ROR of Map (a).

No	Coordinate	ROR	No	Coordinate	ROR
0	(35, 5)	4.2	8	(35, 45)	5.9
1	(65, 5)	4.9	9	(55, 55)	3.5
2	(25, 15)	4.4	10	(75, 55)	5.2
3	(55, 15)	4.2	11	(15, 65)	4.1
4	(5, 25)	3.7	12	(45, 65)	4.3
5	(45, 25)	4.6	13	(35, 75)	5.7
6	(75, 25)	3.8	14	(55, 75)	2.3
7	(15, 35)	5.6	15	(65, 35)	5.9

**Table 2:** Task area coordinates and ROR of Map (b).

No	Coordinate	ROR	No	Coordinate	ROR
0	(65, 5)	4.1	15	(125, 65)	3.8
1	(95, 5)	3.8	16	(65, 75)	3.8
2	(25, 25)	4.3	17	(25.85)	3.8
3	(35, 25)	3.0	18	(45, 85)	4.9
4	(65, 25)	3.9	19	(105, 85)	3.2
5	(95, 25)	3.9	20	(85, 95)	4.6
6	(115, 25)	4.1	21	(125, 95)	4.6
7	(125, 35)	4.1	22	(15, 105)	4.8
8	(15, 45)	4.1	23	(45, 105)	3.1
9	(35, 45)	4.9	24	(95, 105)	4.7
10	(85, 45)	4.6	25	(55, 115)	4.7
11	(65, 55)	4.2	26	(15, 125)	4.4
12	(105, 55)	4.1	27	(75, 125)	5.0
13	(15, 65)	4.8	28	(125, 125)	4.3
14	(35, 65)	4.9			

**Table 3:** Experimental parameter setting.

Parameters	Values
number of UAVs	4
number of task areas	29
max bundle capacity	9
speed of UAV	4 m/s
mission start time	0
mission end time	$ROR_{current}$ $\;<\;0.15\;\times \;ROR_{initial}$
max episode	2000
discount factor	0.95
learning rate	0.01
reward	$R_{total}$
number of neurons per layer	100
memory size	500
batch size	30
number of iterations to replace the target	200

**Table 4:** CBBA task assignment results.

Type of Map	No	Bundle List	Path List	End Time
Map (a)	UAV1	[2, 5, 15, 10, 14]	[2, 5, 15, 10, 14]	31.430
Map (a)	UAV2	[7, 4, 11]	[7, 4, 11]	23.227
Map (a)	UAV3	[0, 3, 1, 6]	[0, 3, 1, 6]	23.942
Map (a)	UAV4	[8, 13, 12, 9]	[8, 13, 12, 9]	28.168
Map (b)	UAV1	[2, 14, 18, 25, 27, 24, 20, 19]	[2, 14, 18, 25, 27, 24, 20, 19]	39.301
Map (b)	UAV2	[9, 11, 10, 12, 15, 21, 28]	[9, 11, 10, 12, 15, 21, 28]	38.952
Map (b)	UAV3	[8, 13, 17, 22, 26, 23, 16]	[8, 13, 17, 22, 26, 23, 16]	37.366
Map (b)	UAV4	[3, 4, 0, 1, 5, 6, 7]	[3, 4, 0, 1, 5, 6, 7]	31.301

**Table 5:** Task area coordinates and ROR.

Type of Map	No	CBBA + IDRL	K-Means + MST	No	CBBA + IDRL	K-Means + MST
Map (a)	0	0.3810	0.3810	8	0.4382	0.3587
Map (a)	1	0.2439	0.4445	9	1.2875	0.3175
Map (a)	2	0.3991	0.2675	10	1.9129	0.3862
Map (a)	3	0.0422	0.2554	11	0.3719	0.2493
Map (a)	4	0.0204	0.3356	12	0.0237	0.3901
Map (a)	5	0.4173	0.4173	13	0.1275	2.0969
Map (a)	6	0.2310	0.3447	14	0.4643	1.0334
Map (a)	7	0.0301	2.0601	15	0.0019	2.1704
Map (b)	0	0.5549	0.5549	15	0.6281	0.7672
Map (b)	1	0.5142	0.344	16	1.3979	0.2310
Map (b)	2	0.0353	0.096	17	0.2822	0.1548
Map (b)	3	0.0033	0.0819	18	0.0492	0.6631
Map (b)	4	0.5278	0.5278	19	1.1772	0.2902
Map (b)	5	0.5278	0.5278	20	0.2797	0.2797
Map (b)	6	0.3719	0.2493	21	0.6225	0.6225
Map (b)	7	1.0110	0.2493	22	0.6496	0.6496
Map (b)	8	0.5548	0.5548	23	0.0380	0.1885
Map (b)	9	0.0897	0.6631	24	0.7769	0.2858
Map (b)	10	0.7603	0.4173	25	0.2858	0.2858
Map (b)	11	0.3810	1.545	26	0.3991	0.5954
Map (b)	12	0.5548	0.3719	27	0.1118	0.1668
Map (b)	13	0.6496	0.6496	28	1.5818	1.5818
Map (b)	14	0.663	1.803			

## Data Availability

Not applicable.

## Nomenclature

**Parameter**	**Definition**
*s*	state of UAV
*a*	action of UAV
*R*	reward function
*d*	euclidean distance
*ρ*	switch for *R*_3_
*k*	discount factor
*π*	strategy of the agent
*p*	action choice probability
*y_i_*	winning score list
*z_i_*	winning agent list
*V*	state value function
*Q*	action value function
*P*	state transition matrix
*e*	revenue function of the searched area
*z*	time
*ε*	search capability of UAV
*α*	learning rate
*b_i_*	bundle of agent
*c_ij_*[*b_i_*]	score function
*L_t_*	maximum assigned task number

## Abbreviations

The following abbreviations are used in this manuscript:

CBBA	Consensus-based bundle algorithm
UAV	Unmanned aerial vehicle
IDRL	Independent deep reinforcement learning
MARL	Multi-agent reinforcement learning
MUTAPP	Multi-UAV target assignment and path planning
TSP	Traveling salesman problem
MTSP	Multiple traveling salesman problem
GA	Genetic algorithm
OPA	Overall partition algorithm
MST	Minimum spanning tree
MEP	Maximum entropy principle
SA	Simulated annealing
MADDPG	Multi-agent deep deterministic policy gradient
ROR	Rate of return
CPP	Coverage path planning
